# Undiagnosed tuberculosis in patients with HIV infection who present with severe anaemia at a district hospital

**DOI:** 10.4102/phcfm.v9i1.1406

**Published:** 2017-06-30

**Authors:** Mbulelo Mntonintshi, Don O’Mahony, Sikhumbuzo Mabunda, Kakia A.F. Namugenyi

**Affiliations:** 1 Department of Family Medicine and Rural Health, Faculty of Health Sciences, Walter Sisulu University, South Africa; 2 Department of Public Health, Faculty of Health Sciences, Walter Sisulu University, South Africa; 3 Department of Surgery, Faculty of Health Sciences, Walter Sisulu University, South Africa

## Abstract

**Background:**

Tuberculosis (TB) is a major cause of severe anaemia in patients with human immunodeficiency virus (HIV) infection in South Africa. However, TB can be difficult to diagnose as it may be extra pulmonary and paucibacillary.

**Aim:**

The aim of this study was to investigate undiagnosed TB in patients with HIV infection and severe anaemia and to identify the optimal investigations for diagnosing TB.

**Setting:**

Mthatha General Hospital, a district hospital.

**Methods:**

The study was a case series.

**Results:**

Haemoglobin levels ranged from 3.6 g/dL to 7.9 g/dL, the mean CD4 count was 176 cells/μL and 80% of patients had a positive TB symptom screen. Forty-three (86%) patients had either clinical or bacteriologically proven TB of whom 33 had pulmonary TB, 34 had extra pulmonary TB and 24 had both types. The diagnostic yield for TB was: chest X-ray (CXR) 91%; ultrasound (US) abdomen pericardium and lower chest 62%; sputum Xpert MTB/RIF 35%; TB blood culture 21% and TB urine culture 15%. Blood and urine cultures did not identify any additional cases over those identified by CXR and US. The laboratory turnaround times were as follows: sputum Xpert, 1.6 days; blood culture, 20 days and urine culture, 28 days. CXR and US were done within one day of initial patient assessment.

**Conclusions:**

The majority of HIV patients with severe anaemia had TB disease, and extra pulmonary TB was as prevalent as pulmonary TB. CXR, US and sputum Xpert were the optimum tests for rapid diagnosis of TB. South African national TB/HIV guidelines should incorporate these specific tests to diagnose TB in patients with HIV and severe anaemia.

## Introduction

Tuberculosis (TB) is the most common cause of death among people living with human immunodeficiency virus (HIV) infection, accounting for about one in three AIDS-related deaths globally.^1^ Early diagnosis and treatment of TB reduces mortality in patients with HIV.^2,3^ However, in patients with HIV infection, TB is frequently undiagnosed. Undiagnosed TB is defined as TB that is only diagnosed during screening, at post-mortem or that is missed during an investigation.^4,5^ Screening of outpatients in South Africa with HIV infection for undiagnosed TB has shown rates of 12% – 20%.^4,6,7^ In resource-limited settings of the Americas, south Asia and sub-Saharan Africa, post-mortem studies show that TB accounts for approximately 40% of HIV-related adult deaths and almost half of these TB cases are undiagnosed at the time of death.^8^ In South Africa, one study showed that 42% of adults (of whom 94% had HIV) who died in hospital had undiagnosed TB^9^ and in another, TB was implicated in 67% of deaths but a third of infections were clinically unsuspected.^5^ TB can be difficult to diagnose in patients with HIV infection, as it is often extra pulmonary, thus requiring special investigations, and if pulmonary, it may be paucibacillary, and chest X-ray (CXR) findings may be different from those of immunocompetent patients.^10,11^

TB in patients with HIV infection can present in many forms, including anaemia.^12^ Anaemia is associated with a high mortality in HIV-infected patients.^13^ Patients with anaemia also have a reduced quality of life and higher health resource utilisation.^14^ Anaemia is an independent predictor of early incident TB among HIV-infected patients in sub-Saharan Africa.^15^ TB chemotherapy combined with antiretroviral therapy results in cure of TB and resolution of anaemia in most patients.^16^

In patients with HIV infection and severe anaemia, defined as a haemoglobin level less than 8 g/dL,^17^ studies suggest that the predominant causes in southern Africa are TB^18^ and anaemia of chronic disease.^12^ South African Department of Health guidelines^19^ recommend additional investigations, including ruling out TB in these patients. However, these guidelines do not specify which investigations to do particularly for extra pulmonary tuberculosis (EPTB) which is as common as pulmonary tuberculosis (PTB) in HIV-infected patients.^20^

The aims of this study were to determine the prevalence of undiagnosed TB in HIV-positive patients with severe anaemia presenting at a district hospital in the Eastern Cape province and to identify the optimal investigations for diagnosing TB in these patients.

## Research methods

### Study design

This study was a prospective case series.

### Setting

The study was conducted in the Emergency and Outpatient Departments of Mthatha General Hospital. The hospital serves an estimated population of 451 712 that is predominantly rural, of low socio-economic status^21^ and with a HIV prevalence of 20% in the reproductive age group (15–49 years) in 2012.^22^

### Study population and sampling procedure

A case was defined as a patient aged 18 years or older with HIV infection and severe anaemia who after treatment by the doctors on duty was not investigated for TB. Patients on TB treatment or who had active or recent haemorrhage (within the last three months) were excluded. A convenience sample of 50 consecutive patients was chosen.

### Operational procedures and data collection

Each patient had an automated full blood count using an ADVIA^®^ 2120 Haematology Analyser (Siemens, Munich, Germany). A symptom screen was undertaken for TB, namely, presence or absence of a cough (of any duration), unintentional weight loss, night sweats and fever^23^ and a physical examination by a researcher. Investigations carried out for the diagnosis of TB were CXR; ultrasound examination (US) of the abdomen, pericardium and lower chest (as per protocol by Heller et al.^24^); Bactec® Mycobacterium TB blood culture; and first void urine TB culture. The CXR was interpreted by a radiologist and by two family physicians. All findings were recorded on prespecified data collection sheets.

A clinical diagnosis of TB was made on clinical evidence without bacteriological confirmation of disease. The clinical diagnosis of TB on CXR was the opinion of the radiologist; and for US examination, the clinical diagnosis was the opinion of family physicians experienced in US examination using the following criteria: for TB abdomen, one or more of: intra-abdominal lymph nodes ≥ 1.5 cm in diameter, hypo-echoic areas 0.5 cm–2.0 cm diameter (consistent with micro-abscesses) in the spleen, and ascites in the absence of clinical evidence of another possible cause.^24^ Patients with pericardial effusion were referred to a specialist cardiology clinic to make a diagnosis of TB pericarditis. A pleural effusion was diagnosed as TB if (1) it was an exudate, (2) cytological examination showed lymphocyte predominance and (3) there were no discordant features (to suggest an alternative diagnosis). Bacteriologically confirmed TB was mycobacterium TB complex identified from a clinical specimen, by Xpert MTB/RIF^®^ assay, smear microscopy or culture. All laboratory tests were performed by the National Health Laboratory Service (NHLS).

### Data analysis

Data were captured and coded in Microsoft Excel 2010 (Microsoft Corporation, Seattle, WA, USA) and analysed using Stata 14.1 (Stata Corp LP, College Station, TX, USA). Numerical data were explored using the Shapiro–Wilk test for distribution. Numerical data that were not normally distributed were reported on using the median and interquartile range and data that were normally distributed were reported on using parametric statistics (mean, range and standard deviation). The two-sample *t*-test (parametric) or the Wilcoxon sum-rank test (non-parametric) was conducted depending on whether the data were normally distributed or not. This was done to compare the equality of two medians or means. Categorical variables were presented using frequency tables and percentages. Differences between two proportions were measured using the two-sample *t*-test of proportions. The level of significance for hypothesis testing was *p* ≤ 0.05. For observer variability, the kappa coefficient was used as an index of agreement between two categorical variables.

### Ethical consideration

Informed written consent was obtained from each participant before inclusion. If TB was diagnosed by the researchers, then patients were started on treatment immediately. The researchers referred patients to a haematologist when further investigations were needed. Ethical approval to conduct this study was obtained from Walter Sisulu University Research Ethics and Bio-safety Committee (protocol number 046/2013) and permission to do the study was obtained from the Eastern Cape Department of Health.

## Results

Fifty consecutive participants were recruited. Of these participants, 72% were females and the remaining 28% were males. The baseline characteristics of participants are shown in Table 1a-c. The median age of 35.5 years for males was higher compared with 32 years for females but the difference was not significant. Table 1a-c also shows that the haemoglobin levels ranged from 3.6 to 7.9 g/dL with no statistical difference between the median Hb of males and females (*p* = 0.692).

**TABLE 1a:** Baseline characteristics of patients.

Sex	*n*	%	95% confidence interval	*p*
Female	36	72	59.6–84.4	< 0.00001
Male	14	28	15.6–40.4	

**TABLE 1b:** Baseline characteristics of patients.

Characteristics	*n*	Interquartile range	Median	*p*
Age (years) by Sex				
Female	36	25–41	32	0.559
Male	14	26–46	35.5	
Total	50	26–42	32.5	
CD4 Count (cells/μL) by Sex				
Female	33	60–156	95	0.558
Male	13	57–190	143	
Total	46	57–180	99.5	
Viral load (copies/mL) by Sex				
Female	20	164.5–793 000	215 725	0.541
Male	6	0–446 893	106 050	
Total	26	138–715 000	193 225	

**TABLE 1c:** Baseline characteristics of patients.

Hb (g/dL) by Sex	*n*	Range	Mean (standard deviation)	*p*
Female	36	3.6–7.9	6.0 (1.1)	0.692
Male	14	3.7–7.7	5.8 (1.3)	
Total	50	3.6–7.9	5.9 (1.2)	

Forty-six participants had CD4 count results. While 25% of females had CD4 counts ≤ 60 cells/μL, 75% of females had CD4 counts ≤ 156 cells/μL. Similarly, for males, 25% had CD4 counts of ≤ 57 cells/μL and only 25% of participants had CD4 counts ≥ 190 cells/μL. Males were, however, found to have higher median CD4 counts (143 cells/μL) as compared with females (95 cells/μL), but this was not statistically significant (*p* = 0.558). Even though 25% of males had a viral load that was not detectable while 25% of females had a viral load that was ≤ 164.5, there was no statistical difference in the median viral loads of males and females (*p* = 0.541).

Anaemia was characterised as follows in 46 patients: normocytic, 63%; macrocytic, 20% and microcytic, 17% (normal ranges mean corpuscular volume [MCV]: females, 78.9 fL–98.5 fL and males, 83 fL–101 fL). In four patients, Hb was diagnosed by a rapid test without a full blood count. The mean of all values for the mean corpuscular haemoglobin concentration (MCHC) was 30 g/d for males and 29 g/dL for females, and was low for both sexes (normal ranges: females 32.7 g/dL–34.9 g/dL and males 31.5 g/dL–34.5 g/dL). The mean Red Cell Distribution Width (RDW) of 20% was high (normal ranges: female 12.4% – 17.3% and males 11.6% – 14%).

### Tuberculosis symptom screen

Of the participants, 40 (80%) had a positive TB symptom screen with one or more symptoms (see Table 2). The majority of participants (64%) were significantly more likely to have been coughing (*p* = 0.005). Unintentional weight loss, night sweats and pyrexia were significantly likely to be found in 92%, 80% and 86%, respectively (*p* < 0.0001).

**TABLE 2:** Tuberculosis symptom screen.

Variable	Yes/No	*n* (%)	*p*
Cough	Yes	32 (64)	0.005
	No	18 (36)	
Unintentional weight loss	Yes	46 (92)	< 0.0001
	No	4 (8)	
Night sweats	Yes	40 (80)	< 0.0001
	No	10 (20)	
Fever	Yes	43 (86)	< 0.0001
	No	7 (14)	

### Physical examination

Six (12%) patients had one or more signs suggestive of EPTB on physical examination, namely lymphadenopathy (4), pleural effusion (2) and meningitis (1). Of these, five had clinically diagnosed or bacteriologically proven TB and one had cryptococcal meningitis.

### Prevalence of tuberculosis

The overall prevalence of TB was 86%: 43 patients (86%) had either or both bacteriological and clinical TB, 42 (84%) had clinical TB and 14 (28%) had bacteriologically proven TB.

### Tuberculosis yield per test

Table 3 shows the yield of TB per diagnostic investigation, that is, the number identified with TB per test. CXR, US and sputum Xpert had the highest yield.

**TABLE 3:** Tuberculosis identified per test.

Test	Number tested	TB identified no. (%)
CXR	32	29 (91)
US	50	31 (62)
Xpert	23	8 (35)
TB blood culture	32	7 (22)
TB urine culture	13	2 (15)

CXR, chest X-ray; TB, tuberculosis; US, ultrasound.

### Time to diagnosis per test

**FIGURE 1:**
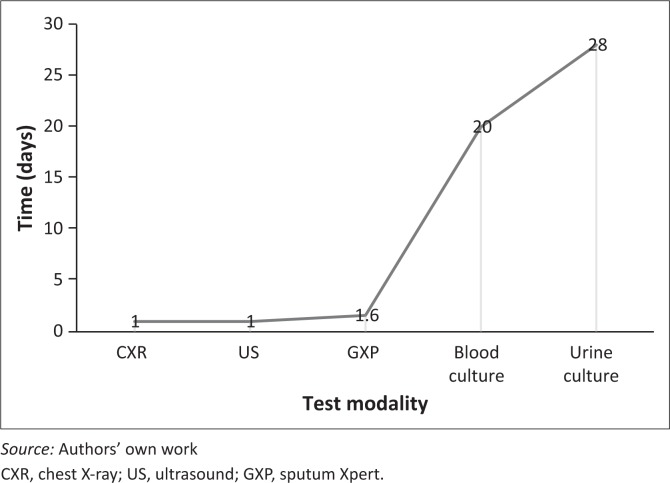
Mean time (days) to tuberculosis diagnosis by test modality.

Figure 1 shows the mean time (days) to diagnosis of TB per test. The tests with the highest diagnostic yield and the shortest period to diagnosis were CXR, US and sputum Xpert.

### Yield from other diagnostic modalities

Of 13 patients who had urine TB cultures, two had positive results. These two patients also had clinical TB on US and one patient had a CXR that also indicated clinical TB. Overall, urine culture did not identify any additional TB cases over those identified by CXR and US. Seven of 30 patients had bacteriologically proven TB on blood culture. All seven had clinical TB on CXR and US. Blood culture did not identify any additional TB cases over those identified by CXR and US.

### Site of tuberculosis

**FIGURE 2:**
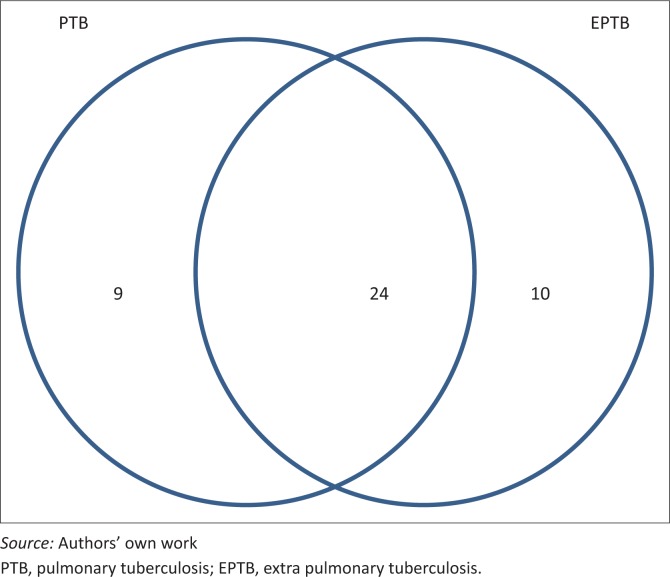
Site of tuberculosis and numbers diagnosed.

Of 43 patients diagnosed with TB, 33 (77%) had PTB, 34 (79%) had EPTB and 24 (56%) had both types. See Figure 2. Of those with EPTB, 31 were identified by US, 2 by CXR (pleural effusions) and 1 by blood culture. US findings were as follows: abdominal TB, 29; combined abdominal TB and pericardial effusion, 1; and pleural effusion, 1.

Seven patients had EPTB on more than one diagnostic modality; of these, one patient had TB on US, blood and urine culture, five patients had TB on US and blood culture and one patient had TB on US and urine culture. Of the three patients with pleural effusions, one had a positive TB blood culture.

### Inter-rater agreement for chest X-ray reading by radiologist and family physicians

Of 32 patients who had a CXR, 29 (91%) had probable TB when assessed by the radiologist. The total CXRs available for comparison by the radiologist and the two family physicians was 31. When the CXRs were assessed by two family physicians, agreement with the radiologist for the presence of TB was 77% for Family Physician 1 and 26% for Family Physician 2. For Family Physicians 1 and 2, the kappa results were, respectively, 0.537 (95% CI 0.162–0.912) representing moderate agreement and 0.0719 (95% CI 0.0183–0.162) representing slight agreement.^25^

## Discussion

A high prevalence (86%) of undiagnosed TB in patients with HIV infection and severe anaemia was found in this study. This finding is consistent with other studies from sub-Saharan Africa. In Malawi, 43% of 77 patients with HIV infection and severe anaemia admitted to a general medical ward had TB (bacteriological and clinical).^12^ In a township in Cape Town, the prevalence of culture confirmed PTB was 40% in 25 patients with HIV infection and severe anaemia.^15^ In a larger study, over a median follow-up of five years of 1521 patients in the same township, TB (pulmonary and extra pulmonary) incidence rates were strongly associated with anaemia severity; those without anaemia had a rate of 4 cases/100 person-years (PYs) compared with 10, 27 and 88 cases/100 PYs in those with mild, moderate and severe anaemia, respectively.^26^ Another study reported severe anaemia in 177 of 575 unselected consecutive HIV-infected adults requiring acute medical admission to a district hospital in Cape Town; of these, 80 (45%) had ‘newly diagnosed TB’ and 14 (8%) had ‘clinical deterioration of known TB’.^18^ Both Malawi and South Africa have a high burden of TB.^27^

Patients in this study were predominantly females (72%) and the proportion in the reproductive age group of 15–49 years was 86%. Similarly, of the patients with severe anaemia and HIV in the study in Cape Town, 59% were females.^18^ The sex and age distribution reflects that of persons with HIV in the South African population where there is a higher overall HIV prevalence in females of 14.4% compared with 9.9% in males, and the proportion of persons aged 15–49 years is 90% (of those aged 15 years or more).^22^ Most patients had advanced HIV disease as indicated by a high viral load and a mean CD4 less than 200, a level below which there is a high risk of severe opportunistic infections and HIV-related cancers.

In this study, 34 (79%) had EPTB. This compares with estimates of more than 50% EPTB in patients with HIV infection in a review by Sharma and Mohan.^20^ Abdominal TB was the most common anatomical site of EPTB (62%), in this study, in contrast to the studies reported by Sharma and Mohan where lymph node TB (35%) was the most common site. In addition, 56% had both PTB and EPTB, indicative of disseminated disease, which is defined as disease that is not limited to one site in the body.^28^ Disseminated TB is common in hospitalised patients with HIV who die in South Africa and was present at autopsy in 100%^5^ and 97% of patients.^29^ Among outpatients with HIV and culture confirmed PTB in Cape Town, 24% had disseminated disease as indicated by the presence of renal disease based on a positive urine lateral flow lipoarabinomannan (LF-LAM) assay.^30^

The optimal combination of tests to rapidly diagnose TB in HIV-infected patients with severe anaemia in this study was CXR, US (abdomen, pericardium and lower chest) and sputum Xpert. These can readily be done in a district hospital. Blood and urine tests did not identify any additional cases over those identified by CXR and US, and they have a lower yield and take longer to obtain results. However, they are important for a bacteriological diagnosis, particularly to exclude drug-resistant TB. An estimated 2.8% of TB cases in South Africa have multidrug-resistant TB (MDR-TB) and 4.9% have isoniazid mono-resistance.^31^ A new urine test, LF-LAM used for rapid diagnosis of TB was not available at Mthatha General Hospital at the time the study was conducted. It is recommended for diagnosis in patients with signs and symptoms of TB with a CD4 count less than or equal to 100 cells/μL or who are seriously ill regardless of CD4 count.^27^

While the CXRs in this study gave a high yield of TB, they were read by a radiologist. In district hospitals, there are no radiologists and X-ray reports are read by general medical officers and family physicians. CXRs in patients with HIV and low CD4 counts can be difficult to interpret as changes from TB can be subtle and atypical.^11,32,33^ Poor inter-rater agreement, as in this study, has been well described in diagnosing TB on CXR depending on the level of experience^34,35^ but can be improved by training.^36^ Training in US is standard in many Family Medicine programmes^37,38^ and there is evidence that family physicians can perform US as well as radiologists.^37,39^ It is, therefore, important to ensure that doctors in district hospitals are trained both in CXR interpretation and US diagnosis of TB in immunocompromised patients.

The results of this study show that 80% of patients had a positive TB symptom screen. However, the doctors who initially assessed the patients did not investigate for TB. National guidelines recommend symptom screening for TB in all HIV-infected patients at every contact with healthcare providers and investigation for TB if there is one or more symptom.^23^ As part of clinical governance, doctors need to adhere to these guidelines. Current national guidelines state that

an Hb < 8 g/dL with no clear cause should generally trigger additional investigations; usually there is an underlying serious OI [*opportunistic infection*], often TB, and this requires urgent diagnosis and treatment.^19^

Based on the high prevalence of TB found in this and other studies, the guidelines should be modified to mandate specific tests to exclude PTB and EPTB.

Six patients had physical signs suggestive of EPTB that were not noted by the doctors who first assessed the patients. This is not unexpected as there is a wide variation in doctors’ ability to discern physical signs and interpret tests.^25^ For example, most studies of the reliability of physical signs such as chest movements, clubbing, vocal fremitus, dullness on percussion and reduced auscultatory percussion have a kappa of less than 0.6, indicating only moderate agreement or less.^40^ It is possible that doctors would have found signs on follow-up examinations.

The majority of patients had a normocytic anaemia. Lewis et al.^12^ reported that anaemia of chronic disease was the most common type of anaemia in HIV-infected patients with severe anaemia in Malawi. In this study, assigning a cause for anaemia was difficult without an extensive haematological workup. All had HIV and the majority had TB. Furthermore, tests were not done for other opportunistic infections that commonly occur with HIV, for example cytomegalovirus and histoplasmosis. However, on the basis of similar studies in southern Africa,^12,18^ it is likely that TB was the underlying cause or precipitant of the severe anaemia.

The MCHC was low for both sexes and is consistent with hypochromia as a result of severe anaemia.^41^ The mean RDW was high and a high RDW has been associated with chronic inflammation, independently of age, sex, MCV, Hb and ferritin in HIV-negative patients^42^; and it has been linked to increased disease activity in HIV infection, manifested by increased viral load and AIDS.^43^

## Strengths and limitations of the study

The results of this study are not generalisable. The sample was non-random. Available results for urine culture were few; this precluded sufficient data for inferential statistical analysis. While there are methods for minimising the impact of missing data, for example, multiple imputation,^44^ the authors did not consider imputing data. Patients were seen only once and other causes of anaemia may have been diagnosed later. A limitation of the study design is the fact that there is no control group and thus limits associations to observations made within the study participants.

The strength of this study is that it has a high clinical relevance on the approach to anaemia in HIV-infected patients in district hospitals.

## Conclusion

The majority of HIV patients with severe anaemia had TB disease and EPTB was as prevalent as PTB. CXR, US (abdomen, pericardium and chest) and sputum Xpert were the optimum tests for rapid diagnosis of TB. It is advised that South African national TB/HIV guidelines incorporate these specific tests to diagnose TB in patients with HIV and severe anaemia.

## References

[1] UNAIDS Global HIV statistics. Fact Sheet November 2016. Geneva, Switzerland: The Joint United Nations Programme on HIV/AIDS (UNAIDS); 2016.

[2] Holtz TH, Kabera G, Mthiyane T, et al Use of a WHO-recommended algorithm to reduce mortality in seriously ill patients with HIV infection and smear-negative pulmonary tuberculosis in South Africa: An observational cohort study. Lancet Infect Dis. 2011;11:533–540. https://doi.org/10.1016/S1473-3099(11)70057-3 21514234 10.1016/S1473-3099(11)70057-3

[3] Peter JG, Zijenah LS, Chanda D, et al Effect on mortality of point-of-care, urine-based lipoarabinomannan testing to guide tuberculosis treatment initiation in HIV-positive hospital inpatients: A pragmatic, parallel-group, multicountry, open-label, randomised controlled trial. Lancet. 2016;387:1187–1197. https://doi.org/10.1016/S0140-6736(15)01092-2 26970721 10.1016/S0140-6736(15)01092-2

[4] Kufa T, Mngomezulu V, Charalambous S, et al Undiagnosed tuberculosis among HIV clinic attendees: Association with antiretroviral therapy and implications for intensified case finding, isoniazid preventive therapy, and infection control. J Acquir Immune Defic Syndr. 2012;60:e22–e28. https://doi.org/10.1097/QAI.0b013e318251ae0b 22627184 10.1097/QAI.0b013e318251ae0b

[5] Wong EB, Omar T, Setlhako GJ, et al Causes of death on antiretroviral therapy: A post-mortem study from South Africa. PLoS One. 2012;7:e47542 https://doi.org/10.1371/journal.pone.0047542 23094059 10.1371/journal.pone.0047542 PMC3472995

[6] Bassett IV, Wang B, Chetty S, et al Intensive tuberculosis screening for HIV-infected patients starting antiretroviral therapy in Durban, South Africa. Clin Infect Dis. 2010;51:823–829. https://doi.org/10.1086/656282 20735240 10.1086/656282 PMC3204934

[7] Lawn SD, Brooks SV, Kranzer K, et al Screening for HIV-associated tuberculosis and rifampicin resistance before antiretroviral therapy using the Xpert MTB/RIF assay: A prospective study. PLoS Med. 2011;8:e1001067 https://doi.org/10.1371/journal.pmed.1001067 21818180 10.1371/journal.pmed.1001067 PMC3144215

[8] Gupta RK, Lucas SB, Fielding KL, Lawn SD Prevalence of tuberculosis in post-mortem studies of HIV-infected adults and children in resource-limited settings: A systematic review and meta-analysis. AIDS. 2015;29:1987–2002. https://doi.org/10.1097/QAD.0000000000000802 26266773 10.1097/QAD.0000000000000802 PMC4568896

[9] Cohen T, Murray M, Wallengren K, Alvarez GG, Samuel EY, Wilson D The prevalence and drug sensitivity of tuberculosis among patients dying in hospital in KwaZulu-Natal, South Africa: A postmortem study. PLoS Med. 2010;7:e1000296 https://doi.org/10.1371/journal.pmed.1000296 20582324 10.1371/journal.pmed.1000296 PMC2889914

[10] Davies P, Pai M The diagnosis and misdiagnosis of tuberculosis. Int J Tuberc Lung Dis. 2008;12:1226–1234. 18926032

[11] Allen CM, Al-Jahdali HH, Irion KL, Al Ghanem S, Gouda A, Khan AN Imaging lung manifestations of HIV/AIDS. Ann Thorac Med. 2010;5:201–216. https://doi.org/10.4103/1817-1737.69106 20981180 10.4103/1817-1737.69106 PMC2954374

[12] Lewis DK, Whitty CJM, Walsh AL, et al Treatable factors associated with severe anaemia in adults admitted to medical wards in Blantyre, Malawi, an area of high HIV seroprevalence. Trans R Soc Trop Med Hyg. 2005;99:561–567. https://doi.org/10.1016/j.trstmh.2005.01.002 15893781 10.1016/j.trstmh.2005.01.002

[13] Belperio PS, Rhew DC Prevalence and outcomes of anemia in individuals with human immunodeficiency virus: A systematic review of the literature. Am J Med. 2004;116(Suppl 7A):27S–43S. https://doi.org/10.1016/j.amjmed.2003.12.010 15050884 10.1016/j.amjmed.2003.12.010

[14] Bolge SC, Mody S, Ambegaonkar BM, McDonnell DD, Zilberberg MD The impact of anemia on quality of life and healthcare resource utilization in patients with HIV/AIDS receiving antiretroviral therapy. Curr Med Res Opin. 2007;23:803–810. https://doi.org/10.1185/030079907X178775 17407637 10.1185/030079907x178775

[15] Kerkhoff AD, Wood R, Vogt M, Lawn SD Predictive value of anemia for tuberculosis in HIV-infected patients in Sub-Saharan Africa: An indication for routine microbiological investigation using new rapid assays. J Acquir Immune Defic Syndr. 2014;66:33–40. https://doi.org/10.1097/QAI.0000000000000091 24346639 10.1097/QAI.0000000000000091 PMC3981888

[16] Kerkhoff AD, Wood R, Cobelens FG, Gupta-Wright A, Bekker L-G, Lawn SD Resolution of anaemia in a cohort of HIV-infected patients with a high prevalence and incidence of tuberculosis receiving antiretroviral therapy in South Africa. BMC Infect Dis. 2014;14:3860 https://doi.org/10.1186/s12879-014-0702-1 25528467 10.1186/s12879-014-0702-1 PMC4300078

[17] World Health Organization Haemoglobin concentrations for the diagnosis of anaemia and assessment of severity. Vitamin and mineral nutrition information system. WHO/NMH/NHD/MNM/11.1. Geneva, Switzerland: World Health Organization; 2011.

[18] Kerkhoff AD, Lawn SD, Schutz C, et al Anemia, blood transfusion requirements and mortality risk in human immunodeficiency virus-infected adults requiring acute medical admission to hospital in South Africa. Open Forum Infect Dis. 2015;2:ofv173 https://doi.org/10.1093/ofid/ofv173 26730391 10.1093/ofid/ofv173 PMC4693115

[19] Department of Health National consolidated guidelines for the prevention of mother-to-child transmission of HIV (PMTCT) and the management of HIV in children, adolescents and adults. Pretoria: Department of Health; 2015.

[20] Sharma S, Mohan A Extrapulmonary tuberculosis. Indian J Med Res. 2004;120:316–353. 15520485

[21] Stats SA Census 2011 Municipal Report Eastern Cape. Report No. 03-01-50. Pretoria: Statistics South Africa; 2012.

[22] Shisana O, Rehle T, Simbayi LC, et al South African national HIV prevalence, incidence and behaviour survey, 2012. Cape Town: HSRC Press; 2014.

[23] Department of Health National tuberculosis management guidelines 2014. Pretoria: Department of Health; 2014.

[24] Heller T, Wallrauch C, Goblirsch S, Brunetti E Focused assessment with sonography for HIV-associated tuberculosis (FASH): A short protocol and a pictorial review. Crit Ultrasound J. 2012;4:21 https://doi.org/10.1186/2036-7902-4-21 23171481 10.1186/2036-7902-4-21 PMC3554543

[25] McGinn T, Wyer PC, Newman TB, et al Tips for learners of evidence-based medicine: 3. Measures of observer variability (kappa statistic). CMAJ. 2004;171:1369–1373. https://doi.org/10.1503/cmaj.1031981 15557592 10.1503/cmaj.1031981 PMC527344

[26] Kerkhoff AD, Wood R, Cobelens FG, Gupta-Wright A, Bekker L-G, Lawn SD The predictive value of current haemoglobin levels for incident tuberculosis and/or mortality during long-term antiretroviral therapy in South Africa: A cohort study. BMC Med. 2015;13:70 https://doi.org/10.1186/s12916-015-0320-9 25889688 10.1186/s12916-015-0320-9 PMC4411796

[27] World Health Organization Global tuberculosis report 2015. Geneva: World Health Organization; 2015.

[28] World Health Organization Consolidated guidelines on the use of antiretroviral drugs for treating and preventing HIV infection. Recommendations for a public health approach. 2nd ed. Geneva, Switzerland: World Health Organization; 2016. 27466667

[29] Martinson NA, Karstaedt A, Venter WD, et al Causes of death in hospitalized adults with a premortem diagnosis of tuberculosis: An autopsy study. AIDS. 2007;21:2043–2050. https://doi.org/10.1097/QAD.0b013e3282eea47f 17885294 10.1097/QAD.0b013e3282eea47f

[30] Kerkhoff AD, Wood R, Vogt M, Lawn SD Prognostic value of a quantitative analysis of lipoarabinomannan in urine from patients with HIV-associated tuberculosis. PLoS One. 2014;9:e103285 https://doi.org/10.1371/journal.pone.0103285 25075867 10.1371/journal.pone.0103285 PMC4116167

[31] National Institute for Communicable Diseases South African tuberculosis drug resistance survey 2012–14. Johannesburg, South Africa: National Institute for Communicable Diseases; 2016.

[32] Perlman DC, El-Sadr WM, Nelson ET, et al Variation of chest radiographic patterns in pulmonary tuberculosis by degree of human immunodeficiency virus-related immunosuppression. Clin Infect Dis. 1997;25:242–246. https://doi.org/10.1086/514546 9332519 10.1086/514546

[33] Huang L Pulmonary Manifestations of HIV [homepage on the Internet]. San Francisco, CA: University of California c2009. [updated 2009 Jan; cited 2016 Dec 21]. Available from: http://hivinsite.ucsf.edu/InSite?page=kb-04-01-05

[34] Reid MJA, Shah NS Approaches to tuberculosis screening and diagnosis in people with HIV in resource-limited settings. Lancet Infect Dis. 2009;9:173–184. https://doi.org/10.1016/S1473-3099(09)70043-X 19246021 10.1016/S1473-3099(09)70043-X

[35] Zellweger J, Heinzer R, Touray M, Vidondo B, Altpeter E Intra-observer and overall agreement in the radiological assessment of tuberculosis. Int J Tuberc Lung Dis. 2006;10:1123–1126. 17044205

[36] Dawson R, Masuka P, Edwards D, et al Chest radiograph reading and recording system: Evaluation for tuberculosis screening in patients with advanced HIV. Inter J Tuberc Lung Dis. 2010;14:52–58. PMC3647461 20003695

[37] Dresang LT, Rodney WM, Rodney KM Prenatal ultrasound: A tale of two cities. J Natl Med Assoc. 2006;98:167–171. 16708502 PMC2595060

[38] Molokwu J Obstetrics and gynecology ultrasound topics in family medicine resident training. Donald School J Ultrasound Obstet Gynecol. 2014;8:31–34.

[39] Keith R, Frisch L Fetal biometry: A comparison of family physicians and radiologists. Fam Med. 2001;33:111–114. 11271737

[40] Benbassat J, Baumal R Narrative review: Should teaching of the respiratory physical examination be restricted only to signs with proven reliability and validity? J Gen Intern Med. 2010;25:865–872. https://doi.org/10.1007/s11606-010-1327-8 20349154 10.1007/s11606-010-1327-8 PMC2896600

[41] Greer JP, Arber DA, Glader B, et al Wintrobe’s clinical hematology. 13th ed. Philadelphia, PA: Lippincott, Williams & Wilkins; 2014.

[42] Lippi G, Targher G, Montagnana M, Salvagno GL, Zoppini G, Guidi GC Relation between red blood cell distribution width and inflammatory biomarkers in a large cohort of unselected outpatients. Arch Pathol Lab Med. 2009;133:628–632. 19391664 10.5858/133.4.628

[43] Gallego ML, Pérez-Hernández IA, Palacios R, et al Red cell distribution width in patients with HIV infection. Open J Internal Med. 2012;2:7–10. https://doi.org/10.4236/ojim.2012.21002

[44] Dong Y, Peng C-YJ Principled missing data methods for researchers. SpringerPlus. 2013;2:222 https://doi.org/10.1186/2193-1801-2-222 23853744 10.1186/2193-1801-2-222 PMC3701793

